# Ambient Air Pollution and Infant Health: Home Monitors Make Cardiorespiratory Connections

**DOI:** 10.1289/ehp.119-a398a

**Published:** 2011-09-01

**Authors:** Kellyn S. Betts

**Affiliations:** Kellyn S. Betts has written about environmental contaminants, hazards, and technology for solving environmental problems for publications including *EHP* and *Environmental Science & Technology* for more than a dozen years.

Children are particularly susceptible to the health effects of air pollution because they spend more time outdoors, have higher respiratory rates, and breathe in a greater volume of air relative to their body weights. Babies may be especially sensitive to the effects of air pollution because their immune, respiratory, and central nervous systems are not fully developed. To date, infants’ respiratory responses to air pollution have been studied much less extensively than those of older children. A new study now links ambient air pollution to an increased risk for apnea (prolonged pauses in breathing) and bradycardia (decreases in heart rate) in babies at high risk for these conditions [*EHP* 119(9):1321–1327; Peel et al.].

The study involved 4,277 infants living in the Atlanta area (about 80 square miles) between 1998 and 2002 whose heart rates and respiration were recorded on home cardiorespiratory monitors. Most of the infants were being monitored because of previous apnea events related to premature birth; others, including some full-term infants, were being monitored for reasons such as gastroesophageal reflux disease. Concentrations of ground-level ozone, nitrogen dioxide, sulfur dioxide, carbon monoxide, oxygenated hydrocarbons, and particulate matter were measured at a centrally located monitoring site.

**Figure:**
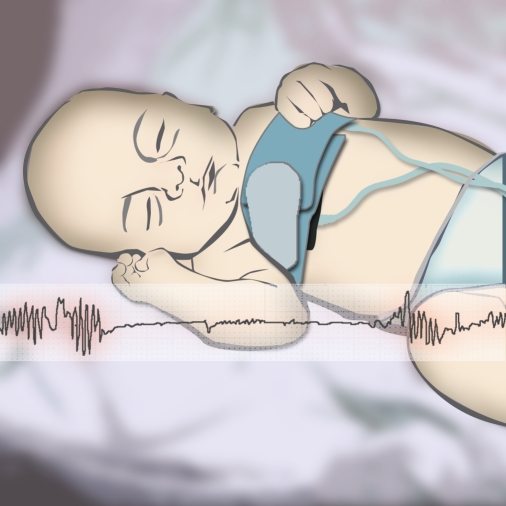
Home cardiorespiratory monitors shed light on health events related to ambient air pollution. Joseph Tart/EHP

The team of researchers documented 8,960 apnea events (in which the child stopped breathing for at least 20 seconds) and 29,450 bradycardia events (in which the child’s heart rate fell below a value determined by his or her age and prematurity status) recorded on the monitors. They examined associations between these events and the 2-day average levels of air pollution, recorded the same day as and the day before each event.

The researchers found significant associations between bradycardia and increases in 8-hour levels of ozone and 1-hour levels of nitrogen dioxide. The relationship between ozone and apnea was similar but not statistically significant. In general, stronger associations were observed for full-term infants with normal birth weights than for premature infants with low birth weights—a surprising but not unprecedented finding. There also was an association between apnea events and increased concentrations of organic carbon in fine particulate matter for full-term, normal-birth-weight infants.

These findings are consistent with previous studies linking air pollution with respiratory symptoms, related hospital admissions, and increased mortality in infants. Although the underlying causes of apnea and bradycardia are unclear, some evidence suggests that immaturity in the autonomic control of the nervous and/or respiratory systems may be involved, which makes a link with increased vulnerability to the effects of air pollution plausible.

